# Impact of Clear Aligners on the Temporomandibular Joint: A Systematic Review

**DOI:** 10.1111/ocr.70094

**Published:** 2025-12-29

**Authors:** Lucca Sicilia, Giza Helen Nonato Miranda, Liana Fattori, Bruno D' Aurea Furquim, David Normando

**Affiliations:** ^1^ Department of Restorative Dentistry, School of Dentistry Universidade Federal de Minas Gerais Belo Horizonte Brazil; ^2^ Department of Orthodontics, Faculty of Dentistry, Post‐Graduation Program of Dentistry Universidade Federal do Pará Belém Brazil; ^3^ Private Practice São Paulo Brazil; ^4^ Private Practice Maringá Brazil

**Keywords:** clear aligner appliances, clear dental braces, invisalign, orthodontic appliances, removable orthodontic appliances, temporomandibular joint

## Abstract

The temporomandibular joint (TMJ) may experience alterations during orthodontic treatments. Furthermore, gaps in the literature highlight a lack of robust evidence on the impact of clear aligner therapy (CAT) on the TMJ. This review aims to find the current evidence regarding the effects of CAT on the TMJ. Electronic searches were conducted in PubMed, Web of Science, Scopus, Embase, LILACS, The Cochrane Library and ProQuest Theses and Dissertations. The eligibility followed PECOS: Permanent‐dentition patients (P); CAT (E); CAT group baseline parameters (C); TMJ structural parameters (O). Data extraction included sample size, age, control group characteristics, assessed outcomes, evaluation tools and it periods and main results. Risk of bias was assessed with the Joanna Briggs Institute checklist and the ROBINS‐E tool. Certainty of evidence was determined using the GRADE approach. Of the 1490 references identified, only five met the eligibility criteria and were included. CAT was associated with dimensional alterations in the condyle volume and surface area, increased superior joint space and fossa depth and decreased condylar bone density. For condylar inclination or position, there are no significant changes. The certainty of evidence was low and very low, reasoned by studies design limitations and heterogeneity. Despite the lower number of studies and low evidence level, CAT seems to induce modest, predominantly increased changes in TMJ spaces and fossa depth, with insufficient clinical significance regarding if CAT effects on TMJ are positive or not. Future studies with standardised TMJ imaging are essential to establish more robust evidence.

**Trial Registration:** PROSPERO CRD42024547484

## 1 Introduction

The temporomandibular joint (TMJ) is a complex structure comprising the mandibular condyle, part of the temporal bone, the articular discs and the articular fossa [1]. TMJ components can suffer alterations through time, which can increase articular eminence inclination and change to a more posterior condylar position [2]. Despite gaps in the literature, it's well established that orthodontic interventions can lead to adaptive changes in these certain components [3].

Modern orthodontics offers a variety of appliances that have significantly evolved with technological advancements [4]. During orthodontic treatment, patients often experience substantial occlusal changes and the mechanics applied by orthodontists can potentially induce structural modifications in the TMJ [3, 5, 6]. These modifications can involve the condyle's positional adaptations and changes in joint loading dynamics, which can influence directly TMJ function and adaptative responses over time, although the long‐term clinical significance of these alterations is still unclear [7]. Additionally, observational studies with short imaging follow‐up reported dimensional changes in joint spaces and fossa depth without strong evidence regarding the long‐term consequences [5, 6].

Orthodontic treatment can be performed using various appliances, such as facial masks, fixed appliances and acrylic removable devices. Depending on the appliance type suggested for the case treatment, it could impact directly on patients' self‐esteem due to aesthetic concerns [8]. Considering that context, the clear aligner therapy (CAT) has emerged as a solution, providing appliances without metallic components and improving patient satisfaction by enhancing self‐esteem and alleviating discomfort [9]. This appliance modality has demonstrated high patient satisfaction and quality of life, directly influencing self‐esteem and the chewing process [10, 11, 12].

Although conventional orthodontic treatment with fixed appliances remains widely used, CAT has proven effective in treating specific types of dental malocclusion [13]. CAT relies on a biomechanical approach determined by the material properties of the device and the custom active surfaces designed by the clinician [14], generating force vectors to achieve appropriate tooth movement while depending on anchoring structures for support [15]. Differently from other appliances, CAT can provide full‐coverage occlusal splinting that can change the patient's vertical dimension and mandibular position when related to maxilla. However, despite its unique biomechanics, some studies suggest that CAT may be associated with dimensional changes in TMJ components, such as the condyle, joint space and overall structural volume [6].

Furthermore, the preliminary studies about the topic have shown heterogeneity in their methodologies, such as eligibility criteria for patients' malocclusion and dentition stage, imagological measures and its findings [3, 5, 6]. Nevertheless, there's no systematic reviews synthesising the evidence regarding the effects of CAT on TMJ, representing a relevant gap for orthodontics. This systematic review aims to evaluate the effects of CAT on the TMJ and synthesise evidence regarding morphological changes caused by CAT in orthodontic patients.

## 2 Material and Methods

### 2.1 Protocol and Registration

This review followed the Preferred Reporting Items for Systematic Reviews and Meta‐Analyses (PRISMA, 2020) guidelines. The study was registered in the PROSPERO database (York, UK) under registration code CRD42024547484. Initially, the present study aimed to review evidence regarding the consequences of CAT on temporomandibular disorders (TMD). However, during the preliminary search, just one eligible study reporting clinical TMD outcomes was identified, due to the lack of studies with that outcome and the search resulting in more anatomical evaluations in TMJ, a change in the PROSPERO protocol was made to provide the current evidence about the morphological consequences on TMJ reasoned by CAT.

### 2.2 Eligibility Criteria

The studies included in this review evaluated the effects of CAT on the TMJ, without restrictions on sex or ethnicity. The acronym PECOS was used to define the eligibility criteria, where Population referred to orthodontic patients with permanent dentition (P), Exposure to CAT (E), Comparators to baseline TMJ parameters of the same CAT group (C), and Outcomes to the effects of CAT on TMJ anatomical structures (O). Although some included studies originally compared CAT with other orthodontic appliances, only CAT groups were analysed in this review to maintain the methodological consistency with the PECOS framework. Review studies, case reports and editorial pages were excluded. Also, studies that did not mention the exposure follow‐up time, studies presenting mixed dentition or without skeletal maturity sample did not match the eligibility criteria.

### 2.3 Search Strategy

The search strategy was developed according to the syntax rules of each database, using a combination of terms and keywords related to orthodontic treatment with clear aligners and the temporomandibular joint, based on the PECOS framework. The search was conducted between January and February 2025 across seven databases: PubMed, The Cochrane Library, Scopus, Embase, LILACS and Web of Science; additionally, grey literature was searched in the ProQuest Theses and Dissertations database using combinations of terms: ‘orthodontic applianc*’; ‘removable orthodontic applianc*’; ‘orthodontic device’; ‘orthodontic retainer*’; ‘orthodontic aligner*’; ‘clear aligner*’; invisalign; ‘temporomandibular joint’; ‘temporomandibular joint disc’; ‘temporomandibular joint disorder*’; ‘temporomandibular joint dysfunction*’ (Table S1). No restrictions were applied regarding the year of publication or language.

### 2.4 Study Selection

All retrieved references were imported into the RAYYAN (Rayyan Systems Inc., 2025) reference manager software [16]. Duplicate citations were manually identified and removed. After duplicates were eliminated, two calibrated reviewers (LS and GHNM) independently selected studies based on predefined eligibility criteria. In cases of disagreement, a third reviewer (DN) was consulted. The selection process was conducted in two phases: initially, studies were screened by title and abstract, and subsequently, full‐text articles of potentially eligible studies were reviewed for final inclusion.

### 2.5 Data Extraction

Data extraction included details such as author, year of publication, study design, sample source, sample size, sex distribution, mean age, malocclusion classification, aligner brand and protocol, treatment duration, TMJ features, evaluation tools and main findings. The variability in malocclusion type and orthodontic protocols was documented and considered potential sources of heterogeneity during evidence synthesis. When necessary, authors were contacted via email, with reminders sent weekly for up to three consecutive weeks. Data were extracted independently by two calibrated reviewers (LS and LF) and then reviewed by the research team (GHNM, BDF and DN) to resolve any discrepancies.

### 2.6 Risk of Bias Assessment

The Joanna Briggs Institute's Critical Appraisal Tool for case series was used to assess the risk of bias [17]. Each checklist included questions with responses of ‘yes,’ ‘no,’ ‘not applicable’ or ‘unclear.’ The assessment range was adapted from Polmann et al. [18] studies with less than 49% ‘yes’ answers categorised as having high risk of bias, those with 50%–69% as moderate risk, and those with 70% or more as low risk. For cohort studies, the Risk of Bias in Non‐Randomised Studies—of Exposure (ROBINS‐E) tool was applied. This tool includes two preliminary sections and seven domains rated as ‘yes,’ ‘probably yes,’ ‘no,’ ‘probably no’ or other similar categories. The overall bias is categorised as ‘very high risk,’ ‘high risk,’ ‘some concerns’ or ‘low risk’ [19]. Two calibrated reviewers (LS and GHNM) did the assessment for both tools and then reviewed by a third reviewer (DN) to resolve any difference in the judgement.

### 2.7 Certainty of Evidence

The certainty of evidence was assessed using the Grading of Recommendations, Assessment, Development and Evaluation (GRADE) approach. Evidence levels were classified as high, moderate or low, based on the study design, quality, accuracy and consistency of the included studies [20].

## 3 Results

### 3.1 Study Selection

**FIGURE 1:**
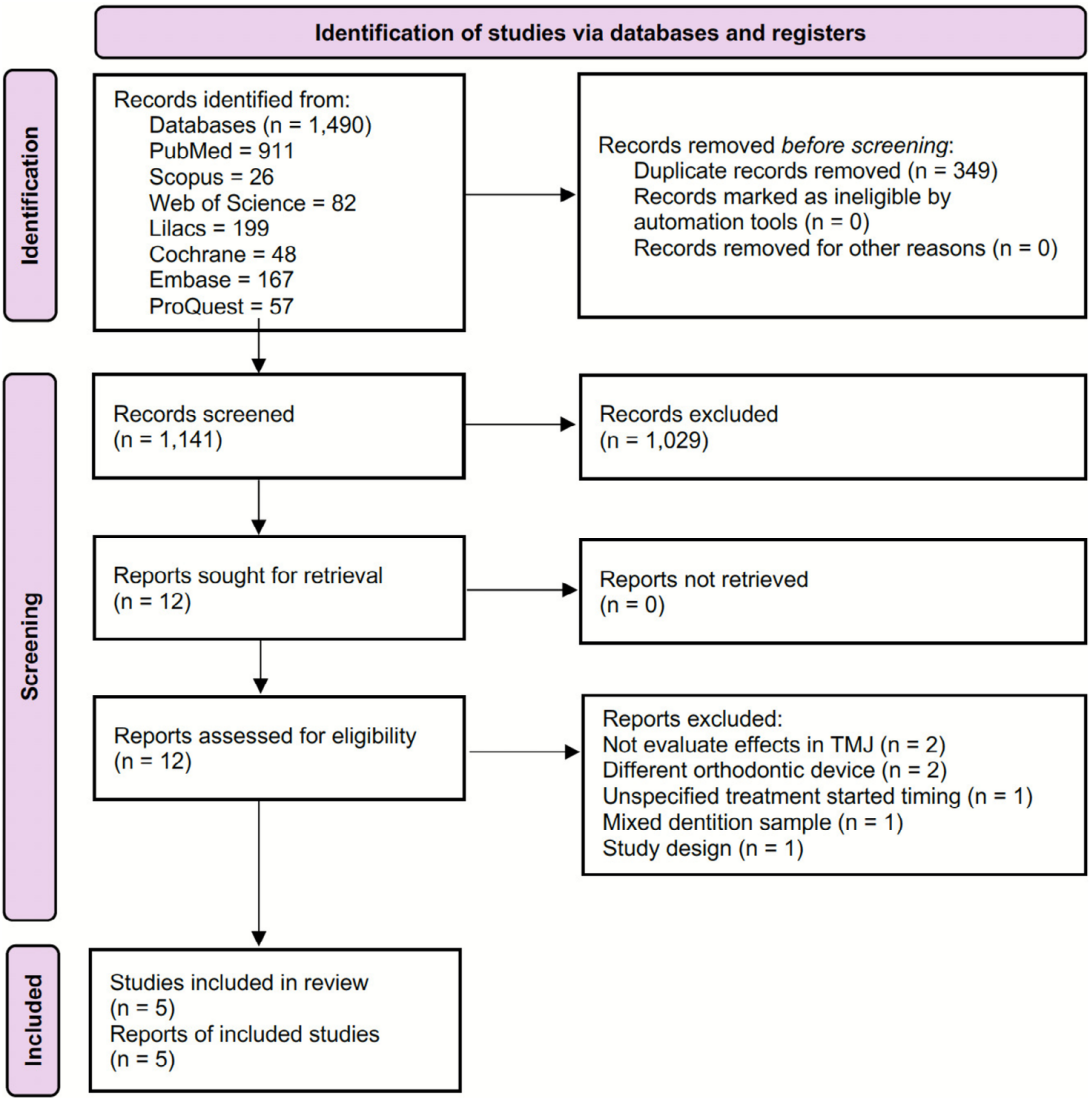
PRISMA flowchart for studies selection.

The searches applied to seven databases identified 1490 references: PubMed (*n* = 911), LILACS (*n* = 199), Embase (*n* = 167), Web of Science (*n* = 82), ProQuest Theses and Dissertations (*n* = 57), The Cochrane Library (*n* = 48) and Scopus (*n* = 26). Duplicates (*n* = 349) were manually removed, leaving 1141 studies for initial screening based on titles and abstracts. In the first selection phase, 1029 records were excluded, and twelve studies were retained for full‐text review. Of these, seven studies were excluded: two did not evaluate TMJ effects, two used alternative orthodontic appliances during treatment, one did not specify the timing of treatment initiation, one showed a different study design and the last evaluated the TMJ parameters in a mixed dentition sample (Table S2). Despite being a limitation reasoned by the lower quantity, five studies met the eligibility criteria and were included in the review (Figure 1).

### 3.2 Study Characteristics

Among the five included studies, four were retrospective cohort studies [3, 6, 21, 22], and one was a before‐and‐after case series [23]. While the cohort studies evaluated changes in the TMJ between aligners and other orthodontic devices, only data related to the aligner group were considered in this review reasoned by the PECOS strategy Comparator (i.e., the baseline TMJ parameters in CAT group).

The present review evaluated data from five studies with an overall of 193 participants. Regarding demographic information, sex was described splitting the participants with 79/39.37% males and 117/60.62% females. The outcomes from the included studies were not stratified for the variable sex.

The mean age of participants varied across studies, ranging from 17.40 ± 2.46 to 29.8 ± 4.6 years. Sample sizes ranged from 23 to 60 participants. Most studies focused on Angle's Class II malocclusion [6, 22, 23], while two assessed patients with mild‐to‐moderate crowding in Class I malocclusion [3, 21]. Treatment durations ranged from 22.82 ± 2.13 to 35.4 ± 11.28 months, though one study did not report treatment duration (Table 1).

**TABLE 1:** Data extracted.

Author (Year)/Type of study	Sample source	Sample size (*n*), M/F (*n*)	Mean age (years)	Malocclusion	Study's aims	Aligner type and protocol	Treatment duration (months)	TMJ evaluation times (T)	TMJ features assessed	Assessment tool	Main results
Al‐Somairi et al. (2025)/CH [21]	Not informed.	*n* = 120–60 for clear aligners group and 60 for fixed appliance group. (52 males and 68 females)	Non‐extracted group 24.27 ± 4.27, extracted group 24.35 ± 4.68	Skeletal Class I	Evaluate the TMJ adaptation after orthodontic treatment with clear aligners and fixed appliances in a sample divided into a four first premolars extracted and a non‐extracted group.	Invisalign.	Non‐extracted group 26.52 ± 8.88, extracted group 35.4 ± 11.28	TMJ was evaluated in two distinct times; T0 = before treatment, T1 = immediately after the treatment ending.	Mandibular fossa dimensions, TMJ spaces and condylar dimensions, inclinations and positions.	The iCat Cone beam computed tomography system was used to take the images, and it was converted for InVivo software.	The inter‐group comparation showed less condylar remodelling after the treatment for clear aligner group. The extraction group anteroposterior condylar position (*p* = 0.014) and anteroposterior condylar joint position (*p* = 0.046) showed statistical difference when compared the mechanics. The intra‐group analyses did not show significant difference between the groups. The findings regarding both clear aligner groups showed a stable condylar posterior position, increase in centric position and decrease in the anterior position.
Al‐Worafi et al. (2024)/CH [22]	Medical University, Stomatological Hospital, Shenyang, Liaoning, China	*n* = 70–35 for clear aligner group (8 males/27 females) and 35 for fixed appliances group (14 males and 21 females)	24.43 ± 7.04	Skeletal Class II and bilateral molar Class II relationship.	Evaluate TMJ dimensions and positions in class II patients using maxillary elastics.	Invisalign and intermaxillary Class II elastics.	24.97 ± 5.17	TMJ was evaluated in two distinct times; T0 = before treatment, T1 = immediately after the treatment ending.	TMJ spaces and condylar dimensions, inclinations and positions.	The iCat Cone beam computed tomography system was used to take the images, and it was converted for InVivo software.	A change in the mediolateral position was observed in both groups, however, that change was less in the clear aligner group. There're not statistical differences between the groups. The author used a percentage analysis to describe the condylar positions. The posterior position showed a lower result in T1, while centric and anterior position presented an increase of these percentage.
Al‐Tayar et al. (2023)/CS [23]	Department of Orthodontics, School of Stomatology, Lanzhou University, Lanzhou, Gansu Province, China.	*n* = 23 (07 males and 16 females)	29.8 ± 4.6	Angle's Class II [six subjects ¼ cusp, nine subjects 1/2 cusp (end‐to‐end) and eight subject's full cusp Class II relationship].	Impact from maxillary molars distalization by clear aligners on the osseous mandibular joint's components and joint spaces on both left and right sides.	Invisalign and intermaxillary Class II elastics.	23.6 ± 7.2	TMJ was evaluated in one time; T1 = immediately after the treatment ending.	Condyle position, condyle‐fossa dimension and TMJ space.	Tomographic 3D analysis performed on In VivoDental, (Anatomage Inc.) software.	No significant differences on mandibular joint's components, joint spaces, mandibular fossa dimensions, mandibular condyle inclination, position, dimension, or volumetric total joint space on both left and right sides of patients before (T0) and after treatment (T1).
Ertugrul & Veli (2022)/CH [3]	Department of Orthodontics, Izmir Demokrasi University Faculty of Dentistry, and Izmir Katip Celebi University Faculty of Dentistry, Izmir, Turkey.	*n* = 28 (14 males and 14 females)	17.40 ± 2.46	Skeletal Class I relationship with mild to moderate crowding.	The effect of orthodontic treatment of individuals with clear aligners (CA) and orthodontic treatment of individuals with conventional brackets (CB) on mandibular condyle bone formation/density.	Invisalign.	Not informed.	TMJ was evaluated in two distinct times; T0 = before treatment, T1 = immediately after the treatment ending.	Condyle bone quality and trabecular bone changes.	Panoramic images with fractal analysis method using the Image J software.	There was a statistically significant reduction in the mandibular condyle bone density in the Clear aligner group (*p* < 0.05) between T0 (14 128 ± 00503) and T1 (14 074 ± 00653).
Zheng et al. (2023)/CH [6]	Department of Orthodontics, School of Stomatology, Air Force Medical University, Xincheng, Xi'an, Shaanxi, China.	*n* = 47 (22 males and 25 females)	23.18 ± 3.76	Skeletal II Class II division 2 malocclusion.	Three‐dimensional analysis of the TMJ before and after orthodontic treatment, and assessment the differences between fixed appliance and clear aligner treatment on the TMJ.	Clear aligner (type not informed).	22.82 ± 2.13	TMJ was evaluated in two distinct times; T0 = before treatment, T1 = immediately after the treatment ending.	Anterior, superior, posterior, medial and lateral joint space; width and depth of the glenoid fossa; sagittal and horizontal condyle angle; height, volume and surface of the condyle.	Tomographic 3D analysis performed on MIMICS software.	Condyle and the surface area of the condyle measurements values showed a statistical significancy (*p* < 0.001) comparing before and after the treatment in the clear aligner group. The anterior joint space decreased, while the superior and posterior joint space, the depth of the glenoid fossa, diameters of the condyle volume and surface area of the condyles increased (*p* < 0.001).

Abbreviations: CH, Cohort study; CS, Case‐Series study; T0, baseline; T1, First TMJ's evaluation after the treatment with clear aligners; TMJ, Temporomandibular joint.

Regarding TMJ features, four studies evaluated morphological parameters using 3D tomographic analysis [6, 21, 22, 23], while one examined bone density patterns using panoramic radiographs [3] (Table 1).

The findings of the present study showed heterogeneity regarding the methodologies, such patients' eligibility criteria, aligner protocol and TMJ assessment tools, denying a possibility of a statistical meta‐analysis.

### 3.3 Results of Individual Studies

Among the five studies, four assessed joint spaces, mandibular fossa dimensions and condylar inclination [6, 21, 22, 23]. Three studies evaluated condylar position [21, 22, 23], while another assessed condylar bone density [3]. Additionally, one study measured condylar volume and surface area [6].

Four studies evaluated joint spaces; one reported statistically significant increases [6], while three found no significant changes after CAT [21, 22, 23]. Similarly, the width and depth of the glenoid fossa significantly increased in one study [6], but three studies reported no significant differences for this variable [21, 22, 23]. Condyle bone density was analysed in one study, revealing a significant reduction (*p* < 0.05) over the treatment period in a sample of 14 patients using clear aligners [3].

Changes in condylar inclination were examined in five studies. Despite differences in study design (four cohort studies and one case series), none showed statistically significant changes in this variable [3, 6, 21, 22, 23].

To provide a comprehensive and transparent overview of these morphological findings, Table 2 summarises the main quantitative outcomes reported by the studies that evaluated their samples, detailing baseline (T0) and post‐CAT (T1), allowing the comparison of the magnitude and direction of changes across studies, providing a clearer understanding of their potential clinical relevance.

**TABLE 2:** Evaluation of morphological parameters of the TMJ before and after treatment with Clear Aligners.

Variable	Al‐Worafi et al. [22]			Al‐Tayar et al. [23]			Al‐Somairi et al. [21]			Zheng et al. [6]		
	T0	T1	*p*	T0	T1	*p*	T0	T1	*p*	T0	T1	*p*
	Mean ± SD	Mean ± SD		Mean ± SD	Mean ± SD		Mean ± SD	Mean ± SD		Mean ± SD	Mean ± SD	
TMJ spaces (mm)												
AJS	2.62 ± 0.69	2.55 ± 0.66	0.354	2.68 ± 1.16	2.78 ± 1.06	0.918	2.55 ± 0.69	2.60 ± 0.76	0.504	2.55 ± 0.35	2.33 ± 0.33	< 0.001*
SJS	3.97 ± 1.05	3.94 ± 0.93	0.779	2.63 ± 0.22	3.19 ± 0.02	0.252	3.91 ± 0.94	3.77 ± 0.97	0.384	2.85 ± 0.44	3.55 ± 0.57	< 0.001*
PJS	2.70 ± 0.71	2.73 ± 0.76	0.773	2.73 ± 1.31	1.79 ± 0.19	0.267	2.88 ± 0.56	2.77 ± 0.46	0.150	1.99 ± 0.37	2.35 ± 0.38	< 0.001*
MJS	4.50 ± 1.50	4.49 ± 1.46	0.902	4.67 ± 0.92	4.36 ± 0.66	0.650	3.29 ± 0.83	3.34 ± 0.92	0.712	2.85 ± 0.50	2.86 ± 0.46	0.857
LJS	—	—	—	—	—	—	—	—	—	2.85 ± 0.42	2.86 ± 0.35	0.461
VTJS (mm^3^)	289.99 ± 53.38	287.45 ± 52.45	0.508	375.06 ± 17.15	358.70 ± 18.98	0.310	290.79 ± 44.56	292.94 ± 48.44	0.685	—	—	—
Mandibular fossa dimension (mm)												
MFH	9.49 ± 1.37	9.48 ± 1.31	0.838	8.19 ± 1.07	7.75 ± 1.08	0.413	11.67 ± 1.44	11.56 ± 2.64	0.753	11.36 ± 0.80	11.70 ± 0.70	< 0.001*
MFW	15.68 ± 1.14	15.69 ± 1.41	0.928	16.17 ± 1.42	18.78 ± 1.93	0.375	17.47 ± 1.67	17.51 ± 1.63	0.603	24.83 ± 2.09	24.85 ± 2.01	0.760
AEH	—	—	—	7.87 ± 1.18	8.05 ± 1.20	0.889	—	—	—	—	—	—
Condylar inclination (°)												
APCI	74.25 ± 4.18	74.38 ± 4.75	0.788	76.86 ± 4.97	76.88 ± 3.99	0.815	70.74 ± 4.83	70.79 ± 4.30	0.914	71.56 ± 3.39	71.73 ± 3.18	0.095
VCI	58.62 ± 8.63	57.25 ± 9.54	0.098	7.04 ± 1.60	7.94 ± 1.98	0.765	58.83 ± 7.16	58.18 ± 6.65	0.423	74.79 ± 2.32	74.74 ± 2.55	0.740
MCI	6.19 ± 3.85	5.97 ± 4.06	0.470	26.38 ± 7.61	24.34 ± 9.57	0.890	10.29 ± 4.47	10.61 ± 5.17	0.629	—	—	—
Condylar position (mm)												
APCP	5.39 ± 1.96	5.23 ± 2.25	0.300	7.34 ± 2.15	7.15 ± 1.55	0.613	7.17 ± 2.37	7.32 ± 2.42	0.532	—	—	—
VCP	10.38 ± 2.32	10.33 ± 2.33	0.724	1.71 ± 0.77	1.55 ± 0.53	0.851	2.36 ± 1.56	2.27 ± 1.36	0.452	—	—	—
MLCP	43.32 ± 2.32	43.44 ± 2.17	2.17	53.24 ± 2.51	52.66 ± 2.39	0.553	44.38 ± 2.40	44.48 ± 2.58	0.215	—	—	—
VCJP	—	—	—	5.64 ± 0.45	5.91 ± 0.33	0.425	6.11 ± 15.04	4.84 ± 16.59	0.596	—	—	—
Condylar dimensions (mm)												
CL	18.23 ± 2.34	18.32 ± 2.40	0.487	18.94 ± 1.16	19.06 ± 1.06	0.946	19.09 ± 1.52	19.15 ± 1.47	0.607	7.03 ± 0.87	7.44 ± 0.91	< 0.001*
CW	12.44 ± 2.10	12.81 ± 2.29	0.104	10.01 ± 0.79	9.27 ± 0.18	0.388	8.05 ± 0.88	8.17 ± 1.19	0.233	16.78 ± 1.40	17.26 ± 1.41	< 0.001*
Height of the condyle (mm)												
	—	—	—	11.75 ± 0.74	12.03 ± 1.12	0.666	—	—	—	7.77 ± 0.75	7.83 ± 0.65	0.181
Volume of the condyle (mm^3^)												
	—	—	—	—	—	—	—	—	—	1574.49 ± 231.82	1681.79 ± 232.35	< 0.001*
Surface area of the condyle (mm^2^)												
	—	—	—	—	—	—	—	—	—	1502.74 ± 223.91	1606.20 ± 239.65	< 0.001*

Abbreviations: AEH, Articular eminence height; AJS, Anterior joint space; APCI, Anteroposterior inclination; APCP, Anteroposterior position; CL, Condylar length (mm); CW, Condylar width (mm); MCI, Mediolateral inclination; MFH, Mandibular fossa height; MFW, Mandibular fossa width; MJS, Medial joint space; MLCP, Mediolateral position; PJS, Posterior joint space; SD, Standard deviation; SJS, Superior joint space; T0, Before treatment; T1, After treatment; VCI, Vertical inclination; VCJP, Vertical condylar joint position; VCP, Vertical position; VTJS (mm^3^), Volumetric total joint space (mm^3^).

* Significant *p* value < 0.05.

### 3.4 Risk of Bias Assessment

**FIGURE 2:**
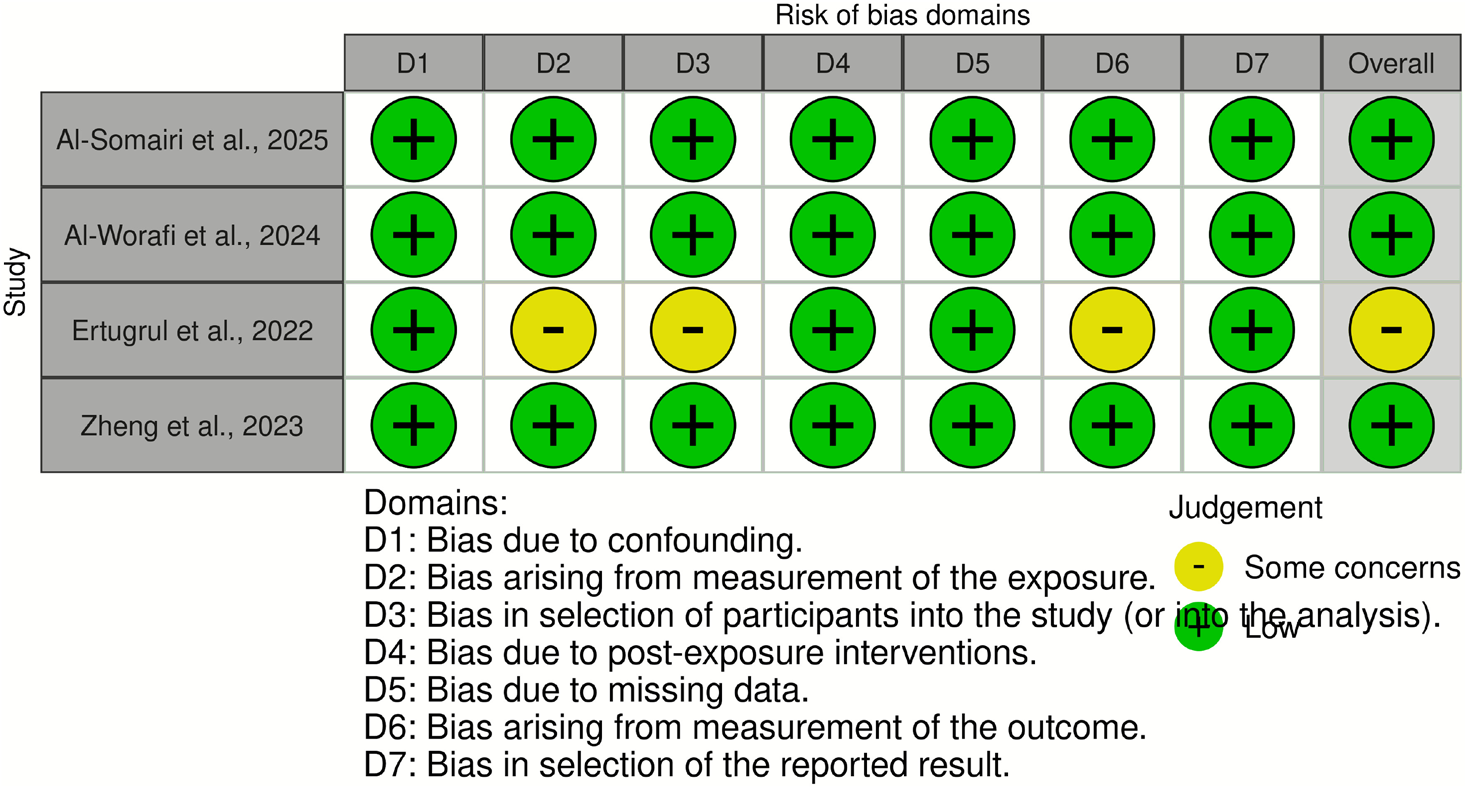
Risk of bias results for cohort studies.

Among the included studies, three demonstrated low risk of bias [6, 21, 22] one presented some concerns [3] and one had moderate risk [23] (Table 3, Figure 2). In the studies with moderate risk or concerns, one case series exhibited statistical biases in test selection [21]. Another study showed concerns regarding exposure measurement, participant selection and outcome assessment [3]. Additionally, one of the studies categorised as low risk did not adequately describe their sample sources, creating demographic gaps in participant data [6].

**TABLE 3:** Risk of bias assessment for case‐series articles.

	Al‐Tayar et al. [23]
Were there clear criteria for inclusion in the case series?	Yes
Was the condition measured in a standard, reliable way for all participants included in the case series?	Yes
Were valid methods used for identification of the condition for all participants included in the case series?	Yes
Did the case series have consecutive inclusion of participants?	Unclear
Did the case series have complete inclusion of participants?	Yes
Was there clear reporting of the demographics of the participants in the study?	No
Was there clear reporting of clinical information of the participants?	Yes
Were the outcomes or follow up results of cases clearly reported?	Yes
Was there clear reporting of the presenting site(s)/clinic(s) demographic information?	No
Was statistical analysis appropriate?	No
Final risk of bias	Moderate

### 3.5 Certainty of Evidence

The analysed outcomes included TMJ morphological parameters and bone density. The certainty of evidence ranged from low to very low due to limitations in study design, risk of bias and imprecision. The case series study [23] presented a moderate risk of bias. Therefore, the GRADE domain ‘risk of bias’ was rated as ‘serious’ for all outcomes assessed in this study (TMJ spaces, mandibular fossa dimensions, condylar inclination, condylar position, condylar dimensions and condylar height). In addition, high variability in standard deviation values was observed for the outcomes TMJ spaces [21, 22] and condylar inclination [21, 22, 23]. Thus, the ‘imprecision’ domain was also rated as ‘serious.’ Mean and standard deviation values of the morphological parameters are presented in Table 2. The outcome mandibular condylar bone density was evaluated in a single study [3] that showed some concerns of bias; consequently, the ‘risk of bias’ domain was also rated as ‘serious’ for this outcome. The outcomes with low certainty were the volume of the condyle and the surface area of the condyle, and these were based on only one cohort study [6]. The remaining evaluated outcomes were classified as having very low certainty. All included studies were observational exposure studies, which justified an initial low certainty of evidence. Inconsistencies were observed among findings regarding TMJ anatomical changes after clear aligner therapy. Although reductions in bone density were reported [3], the certainty of this evidence was classified as very low. A summary of the certainty of evidence is provided in Table 4.

**TABLE 4:** GRADE evidence profile table.

Certainty assessment							Impact	Certainty	Importance
№ of studies	Study design	Risk of bias	Inconsistency	Indirectness	Imprecision	Other considerations			
TMJ spaces									
4	Observational	Serious^a^	Not serious	Not serious	Serious^b^	None	Three cohort studies evaluated joint spaces in 112 patients. Two of these studies reported no statistically significant differences [21, 22]. In contrast, one study found a significant reduction in the anterior joint space and significant increases in the superior and posterior joint spaces [6]. Additionally, a case series involving 23 patients showed no statistically significant differences after treatment with clear aligners [23].	⨁◯◯◯ Very low	Critical
Mandibular fossa dimensions									
4	Observational	Serious^a^	Not serious	Not serious	Not serious	None	Three cohort studies evaluated mandibular fossa dimensions in 112 patients. One study reported a significant increase in glenoid fossa depth [6], whereas the other two cohorts found no statistically significant differences [21, 22]. Additionally, a case series involving 23 patients showed no statistically significant changes after treatment with clear aligners [23].	⨁◯◯◯ Very low	Critical
Condylar inclination									
4	Observational	Serious^a^	Not serious	Not serious	Serious^b^	None	Condylar inclination was evaluated in three cohort studies involving 112 patients, with no statistically significant differences observed after clear aligner therapy [6, 21, 22]. Similarly, a case series including 23 patients reported no statistically significant changes following treatment [23].	⨁◯◯◯ Very low	Critical
Condylar position									
3	Observational	Serious^a^	Not serious	Not serious	Not serious	None	Condylar position was evaluated in two cohort studies and one case series [21, 22, 23], comprising a total of 88 patients. No statistically significant differences were observed following clear aligner therapy.	⨁◯◯◯ Very low	Critical
Condylar dimension									
4	Observational	Serious^a^	Not serious	Not serious	Not serious	None	Condylar dimensions were evaluated in three cohort studies involving 112 patients. One study reported increases in internal, external, anterior and posterior diameters [6], whereas two cohort studies found no statistically significant differences [21, 22]. Additionally, a case series including 23 patients showed no statistically significant changes after clear aligner therapy [23].	⨁◯◯◯ Very low	Critical
Height of the condyle									
2	Observational	Serious^a^	Not serious	Not serious	Not serious	None	Height of the condyle was evaluated in a cohort study involving 47 patients [6] and in a case series including 23 patients [23]. Neither study reported significant changes after treatment with clear aligners.	⨁◯◯◯ Very low	Critical
Volume of the condyle									
1	Observational	Not serious	Not serious	Not serious	Not serious	None	A cohort study involving 47 patients reported a significant increase in condylar volume following clear aligner therapy [6].	⨁⨁◯◯ Low	Critical
Surface area of the condyle									
1	Observational	Not serious	Not serious	Not serious	Not serious	None	A cohort study involving 47 patients reported a significant increase in the surface area of the condyle following clear aligner therapy [6].	⨁⨁◯◯ Low	Critical
Mandibular condyle bone density									
1	Observational	Serious^c^	Not serious	Not serious	Not serious	None	A cohort study evaluated the bone density of the mandibular condyle in 14 patients and showed a significant reduction in this density after treatment with clear aligners [3].	⨁◯◯◯ Very low	Critical

^a^ The case series study presented moderate risk of bias.

^b^ The data showed high variability in the standard deviation value (above 20% of the mean).

^c^ The study presented some concerns of bias.

## 4 Discussion

### 4.1 Summary of Evidence

This systematic review aimed to investigate the effects of clear aligner therapy on TMJ components. The results suggest changes in the position and anatomy of temporomandibular structures, as well as alterations in condylar bone density after the use of clear aligners. However, the studies showed high methodological heterogeneity, lacked control for confounders and presented evidence levels ranging from low to very low. Therefore, these findings should be interpreted with caution.

In line with the PECO strategy, this review included articles evaluating the TMJ before and after treatment with clear aligners. Although some studies compared the effects of aligners with other orthodontic appliances, it was not possible to perform a comparative analysis due to the variability in appliances used across studies, which limited comparisons of aligners with other devices.

Regarding the evaluation of anatomical aspects and condylar position, the studies used three‐dimensional analyses supported by computed tomography, which is considered the gold standard for assessing morphological changes in the TMJ [24]. However, only one study provided information about the patient's posture during imaging [22], even after email requests for clarification. This omission is significant since proper positioning—such as maintaining a closed mouth with the teeth in maximum intercuspation—is crucial for reliable assessment of TMJ spatial relationships [25]. Without this position, measurements may lack accuracy [26].

### 4.2 Bone Density

One study evaluated bone density using the fractal analysis method on panoramic radiographs. Fractal analysis is used to examine complex, self‐similar structures, presenting challenges to conventional evaluation methods [27]. The results are quantified as fractal dimensions [28], a method widely employed to identify changes in the trabecular structure of bone tissues [27, 29, 30]. However, when conducted using panoramic radiographs, the technique faces limitations due to lower image resolution, which may obscure finer bone structures and falsely increase trabecular diameter [31, 32]. Consequently, panoramic radiographs may underestimate fractal dimensions by creating images with an artificially thicker trabecular appearance [31].

Computed tomography (CT) is the ideal method for evaluating TMJ bone density, as it allows for high‐resolution, three‐dimensional assessment without magnification or superimposition [33]. CT can also facilitate fractal measurements in specific sections of varying thickness, enhancing accuracy [28]. Given the methodological limitations of the panoramic radiographs used in the reviewed study, it remains unclear whether clear aligners influence bone density.

### 4.3 Morphological Changes in TMJ Components

Four studies evaluated TMJ morphology before and after aligner therapy in adult patients with Class I [21] and Class II malocclusion [6, 22, 23]. Common parameters included condylar dimensions, condylar position, articular fossa dimensions and joint spaces. Despite efforts to compare findings, methodological differences such as variations in study design and the use of different aligner types prevented a meta‐analysis.

Three studies assessing molar distalization mechanics with aligners found no significant changes in TMJ parameters [21, 22, 23]. The absence of changes was attributed to the lack of molar extrusion and clockwise occlusal plane rotation, which likely prevented premature contacts and TMJ structural modifications. However, these studies did not control for confounders, such as the use of intermaxillary elastics and one of them lacked a control group due to its case‐series design [23], reducing evidence quality.

Conversely, one retrospective cohort study reported changes in joint spaces, increased articular fossa depth, and larger condylar dimensions, volume and surface area [6]. These findings were observed in adults using conventional aligners [6]. The increased superior joint space and fossa depth were attributed to the occlusal splint effect of aligners, which, with a thickness of approximately 0.77 mm [34], promoted prolonged occlusal space opening and mandibular repositioning [35]. Additionally, aligners appeared to facilitate downward and forward condylar movement, like Class II treatment with fixed or functional appliances [36, 37].

Regarding condylar dimensions, one study observed increases in internal and external diameters, anterior and posterior diameters, volume and surface area, suggesting that clear aligners may promote adaptive remodelling [6]. However, these findings may reflect natural growth rather than aligner‐specific effects, particularly given the age of the participants.

Furthermore, the observed changes could be triggered by confounders such as daily time of using the aligners, the necessity of intermaxillary elastics in Class II cases and the individual characteristics of each patient included in the samples, such as format of articular fossa, articular disc thickness and functional factors like chewing, symmetry of mandibular movements and the malocclusion classification.

Identifying TMJ morphological changes with aligners may guide appliance selection, particularly for patients with pre‐existing temporomandibular dysfunction. Although findings were inconsistent and some studies had methodological issues, this review underscores the importance of using current evidence to inform clinical decisions and highlights gaps for future research.

### 4.4 Limitations

None of the included studies assessed signs or symptoms of TMD as outcomes, nor functional aspects of the TMJ. Consequently, these aspects could not be addressed in this review, highlighting a gap for future studies. Additionally, TMJ morphological changes were evaluated using case series and retrospective studies, both of which are prone to confounding factors that may affect results.

The lack of evidence can be strongly associated with the few numbers of studies presented in the literature, as presented in this review with just five studies to meet the eligibility criteria.

Regarding the study's methodology, several questions were raised about the lack of complete information, prompting us to contact the authors for clarification. In the study by Ertugrul and Veli [3], references 42, 43 and 44 were notably absent from the manuscript. We attempted to contact the corresponding author via email three times but received no response.

After not receiving a response from Al‐Worafi et al. [22], we contacted another author from the same study. This time, we successfully obtained clarification regarding the gender distribution within the sample.

Regarding Al‐Somairi et al. [21], we contacted the corresponding author through email asking about the demographic data about sex, but we did not receive a reply.

### 4.5 Clinical Implications

Current evidence suggests that clear aligner therapy may be associated with changes in TMJ morphology and bone characteristics; however, the certainty of evidence is low to very low. Clinically, this highlights the need for careful TMJ assessment before and during orthodontic treatment, particularly in patients with Class II malocclusion or complex biomechanics.

Observed TMJ changes may not be exclusively attributable to aligner therapy itself but may also be influenced by confounders, such as growth status, duration of aligner wear, use of intermaxillary elastics and individual anatomical and functional characteristics of the temporomandibular joint. Therefore, clinicians should interpret TMJ findings cautiously and individualise treatment planning.

At present, the available evidence does not support definitive clinical recommendations favouring clear aligners or conventional fixed appliances based solely on TMJ outcomes. Until higher‐quality studies are available, an evidence‐informed and patient‐centered approach that prioritises both orthodontic objectives and TMJ health is recommended.

### 4.6 Future and Recommended Research

The relationship between clear aligner therapy and TMJ effects, including temporomandibular disorders, remains unclear. Further studies with continuous and standardised evaluation are needed to clarify the effects of clear aligners on adjacent structures, particularly in comparison with conventional fixed appliances, which have been more extensively investigated. In this context, future research should evaluate TMJ outcomes in patients with Class II malocclusion treated with en masse retraction assisted by miniscrews, avoiding the confounding influence of intermaxillary elastics to better isolate treatment‐related effects.

Additionally, the use of advanced imaging modalities, such as magnetic resonance imaging, may enhance the assessment of soft tissues and joint dynamics, contributing to a more comprehensive understanding of the impact of orthodontic treatments on TMD pathology. These findings may have important clinical implications for treatment selection, especially in patients with pre‐existing temporomandibular disorders, supporting orthodontic strategies that prioritise both occlusal correction and TMJ health.

## 5 Conclusions

Although changes in bone density and the morphology of TMJ structures have been suggested in patients treated with clear aligners, the findings among the included studies were inconsistent. Furthermore, the certainty of evidence for the evaluated outcomes ranged from low to very low. Randomised clinical trials and controlled longitudinal studies are essential to generate more robust evidence on this topic.

## Author Contributions

Conceptualization: Lucca Sicilia and David Normando; methodology: Lucca Sicilia, Giza Helen Nonato Miranda and Liana Fattori; supervision: Bruno D'Aurea Furquim and David Normando; writing – original draft: Lucca Sicilia, Giza Helen Nonato Miranda and Liana Fattori; writing – review and editing: Lucca Sicilia, Giza Helen Nonato Miranda and David Normando. All authors agreed to the published version of the manuscript.

## Funding

The authors have nothing to report.

## Conflicts of Interest

The authors declare no conflicts of interest.

## Supporting information


**Table S1:** Search strategy applied.
**Table S2:** Studies excluded after full text evaluation with the reasons (*n* = 6).

## Data Availability

All data analysed in the present review are included in the published articles reviewed and in Appendix S1.
