# Supplementary material for: Impact of Clear Aligners on the Temporomandibular Joint: A Systematic Review
Source: Orthod Craniofac Res. 2025 Dec 29;29(2):209–24. doi: 10.1111/ocr.70094 (PMC12972227; doi:10.1111/ocr.70094)
Supplement: Supplementary file 1 — Table S1: Search strategy applied. Table S2: Studies excluded after full text evaluation with the reasons (n = 6). [file OCR-29-209-s001.docx]

**APPENDIX**

**Supplementary Table 1: Search strategy applied.**

| **Database** | **Search format** |
| --- | --- |
| **PUBMED**  **(n=911)** | ("orthodontic applianc*"[All Fields] OR "removable orthodontic applianc*"[All Fields] OR "orthodontic device*"[All Fields] OR "orthodontic retainers"[All Fields] OR "orthodontic aligner*"[All Fields] OR "clear aligner*"[All Fields] OR ("orthodontic appliances, removable"[MeSH Terms] OR ("orthodontic"[All Fields] AND "appliances"[All Fields] AND "removable"[All Fields]) OR "removable orthodontic appliances"[All Fields] OR "invisalign"[All Fields])) AND ("temporomandibular joint"[All Fields] OR ("temporomandibular joint disc"[MeSH Terms] OR ("temporomandibular"[All Fields] AND "joint"[All Fields] AND "disc"[All Fields]) OR "temporomandibular joint disc"[All Fields]) OR "temporomandibular joint disorder*"[All Fields] OR "temporomandibular joint dysfunction*"[All Fields]) |
| **EMBASE**  **(n=167)** | **#1 'orthodontic applianc*'** OR **'removable orthodontic applianc*'**:ti,ab,kw OR **'orthodontic device'**:ti,ab,kw OR **'orthodontic retainer*'**:ti,ab,kw OR **'orthodontic aligner*'**:ti,ab,kw OR **'clear aligner*'**:ti,ab,kw OR **invisalign**:ti,ab,kw  **#2 'temporomandibular joint'**/exp OR **'temporomandibular joint'** OR **'temporomandibular joint disc'**:ti,ab,kw OR **'temporomandibular joint disorder*'**:ti,ab,kw OR **'temporomandibular joint dysfunction*'**:ti,ab,kw  **#3** #1 AND #2 |
| **WEB OF SCIENCE**  **(n=82)** | 1. **"orthodontic applianc*"** (Topic) 2. **"removable orthodontic applianc*"** (Topic) 3. **"orthodontic device"** (Topic) 4. **"orthodontic retainer*"** (Topic) 5. **"orthodontic aligner*"** (Topic) 6. **"clear aligner*"** (Topic) 7. **Invisalign** (Topic) 8. **"temporomandibular joint"** (Topic) 9. **"temporomandibular joint disc"** (Topic) 10. **"temporomandibular joint disorder*"** (Topic) 11. **"temporomandibular joint dysfunction*"** (Topic) 12. **#1 OR #2 OR #3 OR #4 OR #5 OR #6 OR #7** 13. **#8 OR #9 OR #10 OR #11** 14. **#12 AND #13** |
| **THE COCHRANY LIBRARY**  **(n=48)** | #1. (orthodontic applian*"):ti,ab,kw OR ("orthodontic retainer*"):ti,ab, kw OR ("orthodontic aligner*"):ti,ab, kw OR ("clear aligner*"):ti,ab,kw OR (Invisalign):ti,ab,kw  #2. ("temporomandibular joint"):ti,ab.kw OR ("temporomandibular joint disc"):ti.ab.kw OR ("temporomandibular joint disorder*"):ti,ab.kw OR ("temporomandibular joint dysfunction*"):ti,ab.kw  #3. #1 AND #2 |
| **SCOPUS**  **(n=26)** | 1. TITLE-ABS-KEY ( "orthodontic applianc*" ) 2. TITLE-ABS-KEY ( "removable orthodontic applianc*" ) 3. TITLE-ABS-KEY ( "orthodontic device" ) 4. TITLE-ABS-KEY ( "orthodontic retainer*" ) 5. TITLE-ABS-KEY ( "orthodontic aligner*" ) 6. TITLE-ABS-KEY ( "clear aligner*" ) 7. TITLE-ABS-KEY ( invisalign ) 8. TITLE-ABS-KEY ( "temporomandibular joint" ) 9. TITLE-ABS-KEY ( "temporomandibular joint disc" ) 10. TITLE-ABS-KEY ( "temporomandibular joint disorder*" ) 11. TITLE-ABS-KEY ( "temporomandibular joint dysfunction*" ) 12. ( TITLE-ABS-KEY ( "orthodontic applianc*" ) ) OR ( TITLE-ABS-KEY ( "removable orthodontic applianc*" ) ) OR (TITLE-ABS-KEY ( "orthodontic device" ) ) OR ( TITLE-ABS-KEY ( "orthodontic retainer*" ) ) OR ( TITLE-ABS-KEY ("orthodontic aligner*" ) ) AND ( TITLE-ABS-KEY ( "clear aligner*" ) ) OR ( TITLE-ABS-KEY ( invisalign ) ) 13. (TITLE-ABS-KEY ( "temporomandibular joint" ) ) OR ( TITLE-ABS-KEY ( "temporomandibular joint disc" ) ) OR (TITLE-ABS-KEY ( "temporomandibular joint disorder*" ) ) OR ( TITLE-ABS-KEY ( "temporomandibular joint dysfunction*" ) ) 14. (( TITLE-ABS-KEY ( "orthodontic applianc*" ) ) OR ( TITLE-ABS-KEY ( "removable orthodontic applianc*" ) ) OR (TITLE-ABS-KEY ( "orthodontic device" ) ) OR ( TITLE-ABS-KEY ( "orthodontic retainer*" ) ) OR ( TITLE-ABS-KEY ("orthodontic aligner*" ) ) AND ( TITLE-ABS-KEY ( "clear aligner*" ) ) OR ( TITLE-ABS-KEY ( invisalign ) ) ) AND ( (TITLE-ABS-KEY ( "temporomandibular joint" ) ) OR ( TITLE-ABS-KEY ( "temporomandibular joint disc" ) ) OR ( TITLE-ABS-KEY ( "temporomandibular joint disorder*" ) ) OR ( TITLE-ABS-KEY ( "temporomandibular joint dysfunction*" ) )) |
| **LILACS**  **(n=199)** | (("orthodontic applianc*") OR ("removable orthodontic applianc*") OR ("orthodontic device") OR ("orthodontic retainer*") OR ("orthodontic aligner*") OR ("clear aligner*") OR (invisalign)) AND (("temporomandibular joint") OR ("temporomandibular joint disc") OR ("temporomandibular joint disorder*") OR ("temporomandibular joint dysfunction*")) |
| **PROQUEST**  **Theses & Dissertations**  **(n=57)** | "orthodontic applianc*" OR "removable orthodontic applianc*" OR "orthodontic device" OR "orthodontic retainer*" OR "orthodontic aligner*" OR "clear aligner*" OR invisalign AND "temporomandibular joint" OR "temporomandibular joint disc" OR "temporomandibular joint disorder*" OR "temporomandibular joint dysfunction*" |

**Supplementary Table 2: Studies excluded after full text evaluation with the reasons (n = 6)**

| Excluded Article | Reason |
| --- | --- |
| (Koide, Yamada, Yamaguchi, Kageyama & Taguchi, 2017) | 2 |
| (Nedwed & Miethke, 2005) | 1 |
| (Pittar, Firth, Bennani & Farella, 2023) | 1 |
| (Uzunçibuk, Marrapodi, Meto, Ronsivalle, Cicciù & Minervini, 2024) | 3 |
| (Zhang *et al.,* 2024) | 4 |
| (Zhang *et al.,* 2024) | 5 |
| (Zhu *et al.,* 2022) | 2 |

Reasons for exclusion: 1 – Does not evaluate the effects in temporomandibular joint; 2 – Different orthodontic device used in the sample; 3 – Unspecified beginning time of the treatment for the sample; 4 – Different study design; 5 – Evaluated the outcomes in a mixed dentition sample.

**REFERENCES**

1. Koide D, Yamada K, Yamaguchi A, Kageyama T, Taguchi A. Morphological changes in the temporomandibular joint after orthodontic treatment for Angle Class II malocclusion. Cranio. 2018 Jan;36(1):35-43. doi: 10.1080/08869634.2017.1285218. Epub 2017 Feb 15. PMID: 28198654.
2. Nedwed V, Miethke RR. Motivation, acceptance and problems of invisalign patients. J Orofac Orthop. 2005 Mar;66(2):162-73. English, German. doi: 10.1007/s00056-005-0429-0. PMID: 15827703.
3. Pittar N, Firth F, Bennani H, Farella M. The effect of passive clear aligners on masticatory muscle activity in adults with different levels of oral parafunction. J Oral Rehabil. 2023 Dec;50(12):1409-1421. doi: 10.1111/joor.13575. Epub 2023 Aug 24. PMID: 37615269.
4. Uzunçıbuk H, Marrapodi MM, Meto A, Ronsivalle V, Cicciù M, Minervini G. Prevalence of temporomandibular disorders in clear aligner patients using orthodontic intermaxillary elastics assessed with diagnostic criteria for temporomandibular disorders (DC/TMD) axis II evaluation: A cross-sectional study. J Oral Rehabil. 2024 Mar;51(3):500-509. doi: 10.1111/joor.13614. Epub 2023 Dec 2. PMID: 38041596.
5. Zhang M, Liu X, Zhang R, Chen X, Song Z, Ma Y, Jin Z. Biomechanical effects of functional clear aligners on the stomatognathic system in teens with class II malocclusion: a new model through finite element analysis. BMC Oral Health. 2024 Oct 29;24(1):1313. doi: 10.1186/s12903-024-05114-8. PMID: 39472835; PMCID: PMC11523655.
6. Zhang Y, Zheng J, Wu Q, Jiang T, Xiao H, Du Y, Qi Y, Jin Z, Li F. Three-dimensional spatial analysis of temporomandibular joint in adolescent Class II division 1 malocclusion patients: comparison of Twin-Block and clear functional aligner. Head Face Med. 2024 Jan 6;20(1):4. doi: 10.1186/s13005-023-00404-y. PMID: 38184631; PMCID: PMC10770962.
7. Zhu, C., Li, R., Yuan, L. *et al.* Effects of the advanced mandibular spring on mandibular retrognathia treatment: a three-dimensional finite element study. *BMC Oral Health* **22**, 271 (2022). https://doi.org/10.1186/s12903-022
